# Serological characterization of guinea pigs infected with H3N2 human influenza or immunized with hemagglutinin protein

**DOI:** 10.1186/1743-422X-7-200

**Published:** 2010-08-24

**Authors:** Ruth V Bushnell, John K Tobin, Jinxue Long, Stacey Schultz-Cherry, A Ray Chaudhuri, Peter L Nara, Gregory J Tobin

**Affiliations:** 1 Biological Mimetics, Inc. 124 Byte Drive, Frederick, MD 21702, USA; 2 Department of Swine Infectious Diseases, Shanghai Veterinary Research Institute, Shanghai, China; 3 Department of Infectious Diseases, St. Jude Children's Research Hospital, Memphis, TN 38105, USA; 4 Department of Biological Sciences, College of Veterinary Medicine, Iowa State University, Ames, IA 50010, USA

## Abstract

**Background:**

Recent and previous studies have shown that guinea pigs can be infected with, and transmit, human influenza viruses. Therefore guinea pig may be a useful animal model for better understanding influenza infection and assessing vaccine strategies. To more fully characterize the model, antibody responses following either infection/re-infection with human influenza A/Wyoming/03/2003 H3N2 or immunization with its homologous recombinant hemagglutinin (HA) protein were studied.

**Results:**

Serological samples were collected and tested for anti-HA immunoglobulin by ELISA, antiviral antibodies by hemagglutination inhibition (HI), and recognition of linear epitopes by peptide scanning (PepScan). Animals inoculated with infectious virus demonstrated pronounced viral replication and subsequent serological conversion. Animals either immunized with the homologous HA antigen or infected, showed a relatively rapid rise in antibody titers to the HA glycoprotein in ELISA assays. Antiviral antibodies, measured by HI assay, were detectable after the second inoculation. PepScan data identified both previously recognized and newly defined linear epitopes.

**Conclusions:**

Infection and/or recombinant HA immunization of guinea pigs with H3N2 Wyoming influenza virus resulted in a relatively rapid production of viral-specific antibody thus demonstrating the strong immunogenicity of the major viral structural proteins in this animal model for influenza infection. The sensitivity of the immune response supports the utility of the guinea pig as a useful animal model of influenza infection and immunization.

## Background

The most common mammalian model used for influenza virus research, the mouse, is not susceptible to infection with many unadapted human influenza A viruses of the H3N2 serotype and does not shed virus from the respiratory tract. Ferrets and macaques have increased tropisms to many primary influenza isolates but both are expensive to maintain and difficult to house. Based largely on their recapitulation of human disease signs, ferrets have also been used to derive serotyping reagents for assessing antigenic distance between isolates and potential vaccine strains. However, recent reports suggest that ferrets may not faithfully mimic human immune responses, and that serological tests using ferret sera may not accurately assess vaccine strain efficacy [1,2]. Therefore, there is a need to develop additional permissive small animal models of influenza virus infection that exhibit virus shedding. Serial samples collected from such animal models allow the investigator to determine both the titer and duration of virus shedding from individual animals at multiple times without euthanasia. Further characterization of animal models capable of replicating and transmitting unadapted human, avian, and/or swine influenza viruses can be valuable for studying and testing new and improved vaccines, immunobiotics and anti-virals. Two promising alternative animal models, guinea pigs and cotton rats, have recently been investigated for the analysis of human influenza virus and influenza vaccine [3,4]. These studies focus on the guinea pig as a model for influenza.

Guinea pigs have many attractive features for use as an animal model for influenza immunization and infection. Guinea pigs are relatively inexpensive and easy to maintain for larger studies compared to ferrets and simians. They are readily infected with primary isolates of human influenza strains, and have potential uses for virus evolutionary, prophylactic and therapeutic studies [3]. A small number of reports describing experimental infection of guinea pigs with human influenza viruses were published in the 1970 s and 80 s [5-8]. More recently, we and others have advanced the guinea pig model for the study of virus infection and spread and as a vaccine-challenge model [3]. Guinea pigs can be readily infected with human influenza isolates without prior tissue culture or animal adaptation. The infection in guinea pigs appears to be centered largely in the upper naso-respiratory tract and the animals can pass the virus to others via aerosol transmission [9]. A recent study demonstrated acute viral replication and moderate virulence of the highly pathogenic 1918 pandemic and H5N1 viruses in addition to low-pathogenicity avian and human H1N1 viruses in guinea pigs [10].

The overall purpose of the current study was to characterize the immunological responses of guinea pigs infected with H3N2 virus or immunized with HA protein so as to assess the value of a guinea pig model in future immunological assays such as vaccine-challenge studies. Because of the prophylactic properties of HA-derived vaccines, and their relative ease of production, immune responses of this subunit were studied in the guinea pig model. The results support the utility of the guinea pig as a useful animal model of influenza infection and immunization.

## Results

### Infection of Guinea Pigs

Four groups of guinea pigs were chosen, (1) a negative control with no infection, (2) a positive control that received an infection only, (3) a group that was immunized with low dose of recombinant HA protein, and (4) another with high dose. ELISA extinction titers of Group 1, the control group for this serological study, remained negative and unchanged throughout the study.

**Figure 1:**
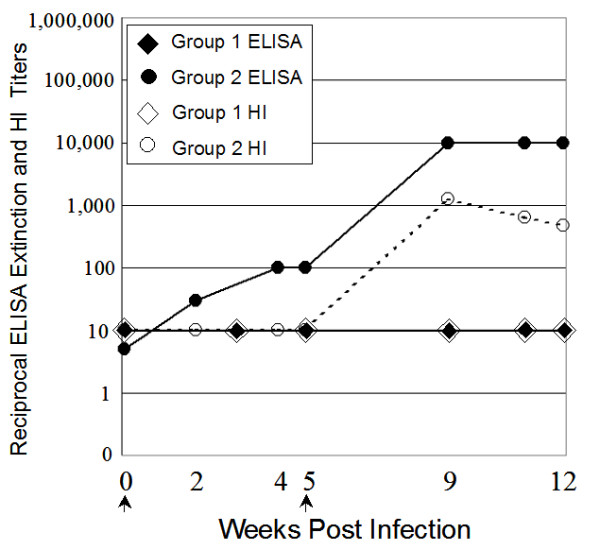
**Analysis of serum pools from infected guinea pigs**. Serum pools were tested for total HA-binding antibodies by reactivity to full-length HA protein in a standard ELISA (solid), and antiviral titers using HI assay (dashed). Arrows along X-axis indicate inoculation dates for Group 2. Error bars for the ELISA extinction titers are shown, but are not readily seen due to their small size.

Two guinea pigs (Group 2) were inoculated intranasally with 3 × 10^4 ^plaque-forming units of A/Wyoming/03/2003 virus, allowed to recover from infection for 5 weeks, and then re-inoculated with the same dose of virus. Nasal wash samples were collected at 2, 3, 6 and 9 days post infection (dpi). The guinea pigs exhibited no outward clinical signs of infection and virus was recovered from nasal washes of each animal between 2 and 6 dpi [3]. Peak titers of progeny virus in this study occurred on day 3 and were in the range of 5 × 10^4 ^and 2 × 10^5 ^pfu/mL of nasal wash fluid (Long et al, in preparation). Serological samples were prepared over the course of the regimen for analysis of total anti-HA antibodies by HA ELISA, antiviral antibodies by HI assay, and identification of linear HA epitopes by PepScan ELISA. Equal volumes of sera from each individual were used to produce pools for each time point in each group. To assess the levels of total HA-specific antibodies, serological samples were assayed by ELISA using plates coated with commercially prepared full-length Wyoming HA glycoprotein (Figure 1). Inoculation with virus and subsequent infection of these guinea pigs resulted in a rise in ELISA titer to the HA protein by the 2^nd ^week which continued to increase through Week 4.

The guinea pigs received a second inoculation of virus on Week 5. Peak virus titers from nasal wash samples occurred again on Day 3 and were determined to be 2 × 10^4 ^and 3 × 10^5 ^pfu/mL for the two animals. Anti-HA ELISA titers rose from 1:100 to 1:10,000 after the second infection with live virus. Antiviral activities in the sera were measured by HI assay (Figure 1). In contrast to the ELISA results, Group 2 HI titers were not detectable 5 weeks after initial infection, and rose only after the re-infection. By Week 9, a significant increase in titer, 64-fold over pre-infected sera, was detectable. In the following three weeks, this peak titer decreased slightly.

### Immunization of Guinea Pigs

**Figure 2:**
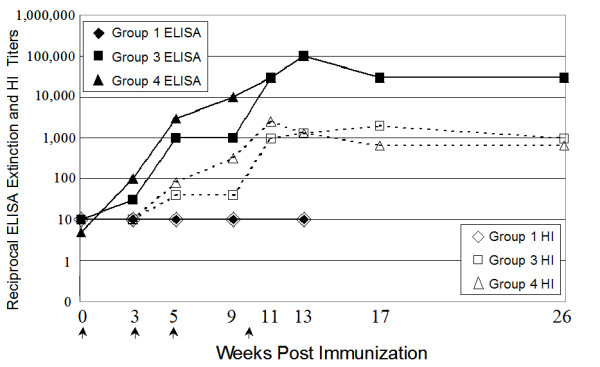
**Analysis of serum pools from immunized guinea pigs**. Sera pools were tested for antibodies that bind to non-denatured full-length HA protein by ELISA and are denoted with solid lines. Sera pools were also tested for antiviral activity by HI, shown with dashed lines. Negative control animals in Group 1 were discontinued after 13 weeks. Arrows along X-axis indicate immunization boost dates. Error bars for the extinction titers are shown, but due to their small size, are not visible.

The two immunized guinea pig groups (Groups 3 and 4) demonstrated similar patterns of increasing antibody titers over the course of the four recombinant HA protein inoculations. Two doses of antigen were initially used to determine the sensitivity of immune reactivity to the HA antigen prior to vaccine-challenge studies with similar subunit antigens. Group 3 (lower antigen dose) ELISA titers initially lagged behind those of Group 4 (higher antigen dose), but caught up after the final boost with equivalent amounts of HA (40 micrograms) in both groups (Figure 2). Interestingly, the ELISA titers persisted at high levels for 4 months following the final immunization and showed little sign of decay. No significant difference was found between the ELISA titers of Groups 3 and 4, with a confidence level of p = 0.33 (ANOVA).

Antiviral HI titers for both groups of HA-immunized animals increased after the second, third, and fourth inoculation (Figure 2). The inflections of the HI titer graphs roughly paralleled the anti-HA ELISA titers throughout the study. After the final boost at week 10, HI titers continued to rise (91- to 128-fold increase over the negative control) and persisted for 16 weeks following the last immunization, with only a 2- to 4-fold drop in magnitude. Sera from Group 1 (negative control) remained negative throughout the study.

**Figure 3:**
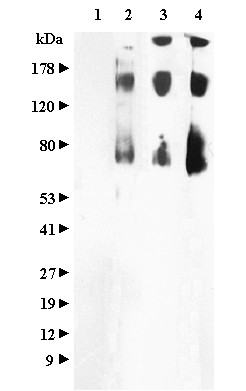
**Western Blot Analysis of sera from immunized and infected Guinea Pigs**. Full-length recombinant HA sera (Protein Sciences, Inc.) was electrophoresed in a denaturing polyacrylamide gel and transferred to membranes. The lanes were cut into strips and probed with Guinea pig sera to confirm the specificity of reactivity. Lane 1: Groups 1 (mock infected) sera, 1:1500; lane 2: Group 2 (influenza infected) sera 1:1500, Lane 3: is Group 3 (immunized with lower concentration of HA protein) sera 1:3000, and Lane 4: Group 4 (immunized with higher concentration of HA protein) sera 1:3000.

The specificity of the immune response to HA protein was assessed using Western blot analysis (Figure 3). Full-length recombinant HA protein was electrophoresed in a denaturing polyacrylamide gel and transferred to nitrocellulose. The membrane was cut into strips and probed with guinea pig sera. Lane 1 shows negative reactivity observed using sera from mock-immunized animals. Lanes 2, 3, and 4 demonstrate serological recognition of HA antigen by animals infected with influenza virus or immunized with purified HA protein. Although the samples were boiled in SDS-buffer containing 2-mercaptoethanol, putative dimeric and trimeric forms of the HA protein are apparent as slower-migrating species.

### PepScan Assays

**Figure 4:**
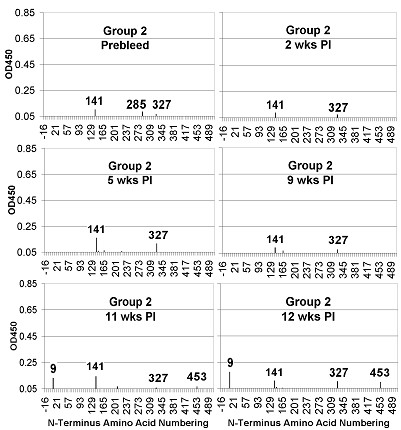
**PepScan ELISA of serum pools from guinea pig infected with influenza virus**. Serum pools (1:750) from Group 2 animals were analyzed for recognition of linear epitopes by reactivity to overlapping peptides bound to microtiter plates. Sequential bleeds were tested from the prebleed (A) and 2 (B), 5 (C), 9(D), 11 (E) and 12 (F) weeks after the initial infection. Reactivity to peptides from sera after infection was compared to the results from the pre-infected sera to identify virus-specific epitopes induced during infection.

To characterize reactivity to linear epitopes, serum pools from sequential bleeds of the infected guinea pigs (Group 2) were tested for binding to a library of overlapping Wyoming HA peptides (Figure 4). Prior to inoculation with virus, the sera showed potential reactivity to Peptides 141, 285, and 327 (Panel A). Peptide 141 is within the A epitope, 285 overlaps the C epitope, and 327 is outside of defined epitopes. Although it was unclear why the pre-infection sera recognized these peptides, reactivity against 141 and 327 remained throughout the study, while reactivity against 285 waned by the second week post-infection. Reactivity against Peptides 9 and 453, both outside of defined epitopes, increased by Week 11 post-infection and was also observed with sera from Week 12 (Panels E and F). Signal strength against 141 and 327 increased in sera from Week 5 post-infection (Panel C), but returned to pre-immune levels by Week 9 (Panel D).

**Figure 5:**
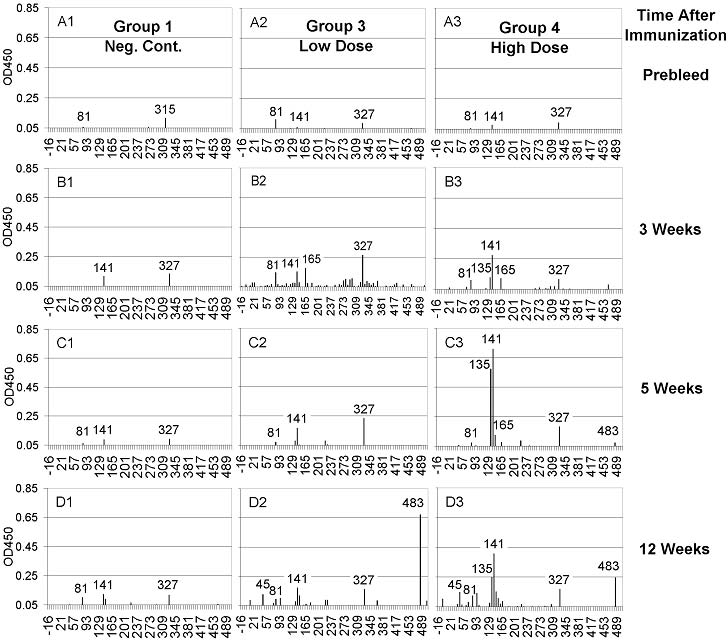
**PepScan ELISA of serum pools from guinea pigs immunized with recombinant HA protein**. Group serum pools (1:750 dilution) were analyzed for recognition of linear epitopes by reactivity to overlapping peptides bound to microtiter plates. The black bars indicate the magnitude of the ELISA reactivity as a measure of Optical Density (O.D.) for the recognition of specific peptides. Sequential bleeds were tested from the prebleed (A) and 3 (B), 5 (C), and 12 (D) weeks after the initial immunization. Reactivity to the peptides was compared between the three groups to identify potential linear epitopes. Group 1: mock immunized negative control group (left column), Group 3: lower dose HA-immunized (center), Group 4: higher dose HA-immunized (right column).

Immune reactivities of sera from HA-immunized guinea pigs were compared with influenza-infected guinea pigs (Figure 5). Sera from mock-immunized animals (Group 1, Panels A1, B1, C1, and D1 of Figure 5) reacted with Peptides 141 and 327, as previously seen with sera from pre-infected guinea pigs (Figure 4A). As the mock-immunized guinea pigs aged, they developed measurable reactivity to Peptide 81, which overlaps the E epitope.

After immunization with lower dose HA antigen, sera from Group 3 animals initially increased overall reactivity against most of the representative peptides in the panel with enhanced reactivity against Peptides 81, 141, 165, and 327 (Panel B2). Immediately prior to the second boost, reactivity against many of the peptides decreased and reactivity primarily against 81, 141 and 327 was seen (Panel C2), which persisted through the study. In addition, after boosting, weak reactivity against Peptide 45, in the C epitope, and strong activity against Peptide 483, outside defined epitopes, were observed (Panel D2).

Prior to inoculations, sera from the higher dose immunization group (Group 4) showed similar low levels of reactivity as seen with the other two immunization groups (Panel A3). At Weeks 3, 5, and 12, sera from Group 4 animals recognized Peptides 81 and 327 with moderate levels of reactivity (Panel B3). Reactivity against Epitope A Peptides 135 and 141 increased in Week 3, peaked in Week 5, and then decreased in Week 12. Similar to what was seen for Group 3, reactivity against Peptides 45 and 483 were observed in later bleeds. PepScan data from serum samples of all groups collected after Week 12 demonstrated patterns of peptide binding similar to those at Week 12 (data not shown). Table 1 contains a summary of the most highly reactive peptides recognized by the guinea pig sera.

**Table 1:** Sequences of Sero-reactive HA Peptides

Peptide N-Terminus	Specificity of Group	Recognized by Pre-immune	Epitope	Amino Acid Sequence
9	Infected	No	none	STATLCLGHHAVPNGTIV

45	Immunized	No	C	SSSTGGICDSPHQILDGE

81	Immunized	Yes	E	NKKWDLFVERSKAYSNCY

135	Immunized	No	A	TSSACKRRSNKSFFSRLN

141	All	Yes	A	RRSNKSFFSRLNWLTHLK

165	Immunized	No	B	NVTMPNNEKFDKLYIWGV

285	Pre-Immune	Yes	C	NGSIPNDKPFQNVNRITY

327	All	Yes	none	QTRGIFGAIAGFIENGWE

453	Infected	No	none	KQLRENAEDMGNGCFKIY

483	Immunized	No	none	NGTYDHDVYRDEALNNRF

### Mapping reactive peptides to 3-D structure

**Figure 6:**
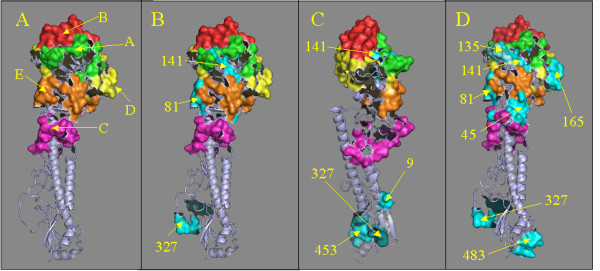
**Peptides recognized by Guinea pig sera localized on the 3D structure**. Panel A shows the monomer structure file of the related H3N2 HA glycoprotein of A/X-31 (H3N2) colorized to identify the locations of the major epitopes A (green), B (red), C (pink), D (yellow), and E (orange). Panel B shows the location of HA peptides that were recognized by negative control guinea pig sera: peptides 81, 141, and 327 (peptides colorized in cyan). Panel C shows peptides recognized by infected Guinea pigs: peptides 9, 141, 327 and 453 (peptides colorized in shades of cyan). Panel D shows peptides recognized by sera from immunized Guinea pig sera: peptides 45, 81, 135, 141, 165, 327 and 483 (peptides colorized in cyan). The structure was drawn from 1HGG.pbd [11] using PyMOL [30].

The position of reactive peptides located on the three-dimensional structure of the related H3N2 strain X-31HA was studied (Figure 6, Panels A-D, 1HGG.pdb, [11]). Figure 6A shows a ribbon diagram of the monomeric ectodomain of HA in which residues in epitopes A-E have been colorized. Figure 6B identifies the locations of peptides 141 and 327, which were seen in pre-infected and mock immunized sera. Peptide 141 contains amino acid residues previously mapped to epitope A (142-146, 150, 152) [12] while peptide 327 is located in a membrane-proximal position, a previously undefined as an area of antigenic interest. Figure 6C shows the location of the two peptides identified in PepScans from influenza infected guinea pigs, Peptides 9 and 453. Figure 6D identifies the positions of Peptides 135 (also contained in epitope A) and 483 that were recognized by sera from immunized animals. As can be seen in Figure 6, Peptides 9, 453, and 483 are located in the membrane-proximal stem of the HA glycoprotein in a region previously not noted for containing epitopes.

## Discussion

A major aim of our research group is the development of broadly protective vaccines that stimulate cross-protective immunity against multiple strains of human influenza viruses [13,14]. In the process of developing and testing vaccines for the stimulation of broadened immunity, it is necessary to raise sera in multiple species of animals for analysis of cross-strain antiviral responses. In addition, it would be helpful to assess protection from cross-strain challenge in multiple animal models. Because of the attractiveness of the guinea pig model for infection with influenza, we have characterized the immune responses after infection or immunization of guinea pigs. Here we present an immunological comparison between guinea pigs infected intranasally with an H3N2 virus and those immunized with the homologous HA glycoprotein, an attractive potential subunit vaccine candidate.

Contrasting the serological results of infected and immunized animals provided interesting insights. The data demonstrated that guinea pigs readily seroconvert in response to both intranasal inoculations of virus and immunizations with the same recombinant HA glycoprotein. A rise in binding antibodies (ELISA positive) preceded the development of antiviral antibodies as determined by hemagglutinin-inhibition (HI positive) for both infected and immunized groups of guinea pigs. The initial lag period was followed by strong correlation between the continued elevation of binding and antiviral (HI) antibodies. ELISA titers rose to approximately 1:100 titers after single inoculations with either infectious virus or purified HA antigen. Peak ELISA titers of infected animals reached 1:10,000, while those of immunized animals reached 1:100,000. However, if Groups 3 and 4 had been limited to only two doses, then titers may have more closely matched Group 2. Measurable antiviral titer required a second dose of virus or immunogen. HI titers of both infected and immunized animals reached approximately 1:1000 and decayed slightly over time. The lack of measurable antiviral immune responses observed before the second inoculation of any of the experimental groups may be due to the lower sensitivity of the HI assay, and is not necessarily an indication that the first infection or immunization did not elicit HI responses. Both ELISA and antiviral antibody titers persisted for many weeks following the final infectious innocula or boost with HA protein. Little, if any, decay of ELISA or HI titers were observed through Week 26 following the final HA immunization at Week 10.

A better understanding of the epitopes recognized by the anti-HA antibody responses in this experimental animal model, and how these epitopes compare to the human immune response, could facilitate more rapid advancements in vaccine design. Five dominant epitopes (A-E) of the HA glycoprotein have been previously characterized by both immunological reactivity in humans and animals, and by evolutionary variability in naturally infected humans. A PepScan analysis was conducted to map the linear B cell epitopes and was intended to correlate immunological reactivity with previous data derived in other animals and in humans. Analysis of conformational epitopes recognized by infected and immunized guinea pigs will be the subject of a future study. Previous immunological studies using overlapping peptides to characterize linear epitopes in influenza and other pathogens have had mixed results [14-19]. Although PepScans have identified epitopes in HIV, Measles, SARs, and Borna virus, most prior studies with this type of analysis failed to detect linear epitopes within the HA glycoprotein [20-22]. However, the continued improvements in peptide synthesis suggested that the approach should be revisited and expanded to encompass the entire HA protein. Interestingly, the data from this study identified two immunodominant epitopes, represented by peptides with N-terminal amino acids 141 and 327, which are recognized by both pre-immune and immune sera. While the interpretation of reactivity by pre-immune sera remains open, these results suggest that recognition of viral epitopes is present in the innate repertoire. If so, it is possible that pre-infection recognition plays a role in skewing the immune system towards a more oligoclonal rather than polyclonal response. Induction of an immune response limited to a small set of epitopes may accentuate recognition of immunodominant epitopes that are often present in regions of high genetic variability in Class II pathogens [13]. The ability to take advantage of the propensity of host immune systems to mount strain-specific immune responses largely limited to variable immunodominant epitopes may be a pathogenesis trait that influenza and other viruses have evolved so as to increase fitness on a landscape made more rugged by host immunity.

Serum from the high dose immunization group (Group 4) showed increased reactivity to peptides 141 and 135 (Figure 6) which both represent a highly variable and immunogenic loop of Epitope A [23]. Unexpectedly, reactivities to additional peptides (9, 327, 453, and 483) derived from regions outside of previously defined epitopes, and near the transmembrane domain, were observed after multiple immunizations and two infectious innocula. The amino acid sequences at the cores of these peptides are highly conserved among influenza A strains. The observation of linear epitopes does not preclude the reactivity of the sera to more dominant conformational epitopes that were not detected by this method. However, in a recent study of cross-reactive epitopes in avian influenza serotypes, Meuller et al. identified several linear epitopes in the HA of H4, H5, and H12 through a similar use of overlapping peptide ELISA [24]. We have aligned the sets of peptides used in both studies to determine analogous peptides so that the results can be compared more easily (data not shown). Interestingly, analogues of many of the H3N2 peptides that were recognized in the present study were also recognized by sera against the avian HA glycoproteins. Avian sera recognized analogues to peptides 141 and 327, which were recognized by pre-immune Guinea pig sera. In addition, avian sera also recognized analogues to peptides 9, 453, and 483. The contribution of reactivity to these peptides towards antiviral activities will require further investigation. Future studies have been planned to characterize the PepScan reactivities of sera from humans infected or immunized with influenza A/Wyoming/03/2003.

Overall, the current study has provided valuable immunogenicity data to further characterize immune responses in a relatively new animal model for human influenza infection and vaccination.

## Conclusions

We present an immunological comparison between guinea pigs infected intranasally with an H3N2 virus, A/Wyoming/03/2003, and those immunized with recombinant HA subunit from the homologous strain. Sera from guinea pig treatment groups, collected over a six month period, were compared serologically for changes induced by each treatment: total antibodies were measured by ELISA, antiviral responses by HI assay, and recognized linear epitopes identified by PepScan ELISA.

Results of this study re-enforce and extend previous reports characterizing the infection of guinea pigs following inoculation with unadapted human influenza strains. The infected guinea pigs mounted vigorous immune responses that had antiviral activities as measured by HI assay. Guinea pigs immunized with purified HA protein developed similar antiviral activities. Pepscan data determined that sera from naïve animals recognize a linear epitope in the defined A epitope and another epitope near the fusion or HA cleavage sites. Further studies will be required to determine whether these innate reactivities are also found in sera from naïve humans. If so, it will be important to assess whether these antibodies offer any protective immunity, or are dysregulatory in nature. Pepscan data also demonstrated the reactivity of sera from infected and immunized animals to linear determinants located both within and outside of previously defined major epitopes. The change in PepScan profiles over the course of the immunization and infection regimens appeared to reflect maturation of the humoral immune responses to linear epitopes. By altering the immunogenicity of the most dominant, yet variable, epitopes, it may be possible to refocus the immune response towards more highly conserved epitopes to derive a more broadly cross-protective influenza vaccine [13,14]. Subunit vaccines, along with well-defined animal models for influenza research, have the potential to more rapidly, and accurately guide the development of future vaccines for both seasonal and pandemic influenza outbreaks.

## Methods

### Cells and Virus

Influenza A/Wyoming/03/2003 (H3N2) was obtained from the Center for Disease Control and Prevention. The virus was originally derived by reassortment and contains genes encoding HA and neuraminidase of Wyoming, with all other genes from A/Puerto Rico/8 H1N1 virus [25]. The virus was propagated in monolayer cultures of Madin-Darby canine kidney (MDCK) cells (ATCC #CCL-34) using Dulbecco's Modified Eagle Medium (Lonza), supplemented with 7% fetal bovine serum (Lonza). For plaque assays, virus samples were serially diluted into 1 mL of phosphate buffered saline (PBS) and placed into 6-well plates confluent with MDCK cells. After an 1-hour (h) incubation, the innocula were replaced by a mixture of 1% molten agar in complete growth media. Upon solidification of the agar, the plates were inverted and incubated in a humidified 37°C incubator. Plaques were typically visible for enumeration or isolation 3-4 days after inoculation. Prior to introduction into animals, MDCK propagated virus stocks were titered using a plaque assay and adjusted to 3 × 10^5 ^plaque-forming units/mL (pfu/mL) with sterile saline.

### HA Protein Expression and Purification

Recombinant influenza A/Wyoming/03/2003 hemagglutinin (HA) was produced in stably transformed S2 drosophila cells [26,27]. Briefly, the A/Wyoming/03/2003 gene was subcloned from a parental plasmid vector (a kind gift of Dr. Kanta Subbarao, NIAID, NIH) into pMT-BiP-V5-His (Invitrogen, Inc.) such that the mature ectodomain (amino acids 17-513) was in-frame with the BiP insect cell promoter, and sequences encoding a hexahistadine tract were inserted immediately upstream of a stop codon. S2 drosophila cells were co-transfected with the HA expression plasmid and pCoBLAST (Invitrogen, Inc), and stable transformants selected with blastocidin (30 micrograms/mL, Thermo Fisher Scientific). Expression of recombinant HA protein was induced for four days by the addition of 1 mM cupric sulfate to the culture media. After expression, conditioned supernatants containing the secreted HA protein were clarified at 2,000 × *g *for 20 min. The HA protein was purified through a multi-step process including chromatographies on copper-charged Fast Flow Sepharose (GE Bio) using elution with 50 mM imidazole, lentil lectin agarose (Vector Labs) using elution with 0.5 M alpha-methly-D-mannoside, and, finally, anion exchange in DE53 resin (Whatman) at pH 8.8 with elution in 50-100 mM NaCl. The eluted samples were concentrated and buffers exchanged after each chromatography step using filtration spin-cartridges with 30,000 molecular weight cut-off membranes (Amicon Ultra Centrifugal Filter Devices, Millipore). Protein yield and purity were determined using the Pierce Coomassie Protein assay reagent with a bovine serum albumin standard, and Western blotting with comparison to commercially prepared standards of full-length A/Wyoming/03/2003 HA glycoprotein (a kind gift of Dr. Joseph A. Rininger, Protein Science Corporation). A mock preparation of the HA ectodomain protein was produced using the above expression and purification methods, and stably transformed S2 cells containing the empty pMT-BiP vector lacking the HA gene for use as a negative control in immunization experiments.

### Guinea Pig Infections and Immunizations

Six to eight weeks of age guinea pigs were obtained from Harlan-Spraque-Dowley Inc., and animal studies performed at BioCon Inc, Rockville, MD followed appropriate AAALAC-approved guidelines for the humane treatment of animals in research. Guinea pigs were divided into four groups (Table 2) and test bleeds were collected prior to the study. Group 1 (n = 4, each) guinea pigs were immunized subcutaneously with the mock prepared negative control protein, and served as a negative control. Group 2 (n = 2), were lightly anesthetized and intranasally inoculated with 1 mL of A/Wyoming/03/2003 influenza virus (3 × 10^4 ^pfu/mL). Animals were re-infected at five weeks after the first inoculation with the same dose of virus. Guinea pigs in Groups 3 and 4 (n = 4) were subcutaneously immunized with recombinant HA protein in Complete Freund's Adjuvant (Thermo Fisher Scientific) and boosted at weeks 3, 5, and 10 with HA protein in Incomplete Freund's Adjuvant (Thermo Fisher Scientific) to characterize the boosting effects of the HA antigen. Initial experimental design also included a comparison of increasing antigen load to study how the animals responded to increasing concentrations of antigen. This was an attempt to scale the amount of recombinant HA protein to that which would be presented by natural infection. Animals in Group 3 were immunized three times with 10 micrograms each, and then given a final boost of 40 micrograms at 10 weeks post-prime. Group 4 animals were immunized three times with 30 micrograms of recombinant HA, with a final boost of 40 micrograms. At the same intervals, Group 1 control guinea pigs were immunized with the mock protein preparation derived from the insect cell system used to propagate the HA recombinant antigen.

**Table 2:** Guinea Pig Infection and Immunization Regiments

Group #	Study	Antigen	# of Doses	Week	Dose
1 (n = 4)	Neg. Control	Mock-produced HA empty pMT-BIP	4	0, 3, 5, 10	30 ug, 30 ug, 30 ug, 40 ug (Total Protein)

2 (n = 2)	Infection	A/Wyoming/2003	2	0, 5	3 × 10^4 ^pfu, 3 × 10^4 ^pfu

3 (n = 4)	Immunization (lower Dose)	A/Wyoming/2003 HA ectodomain	4	0, 3, 5, 10	30 ug, 30 ug, 30 ug, 40 ug (Total Protein)

4 (n = 4)	Immunization (Higher Dose)	A/Wyoming/2003 HA ectodomain	4	0, 3, 5, 10	30 ug, 30 ug, 30 ug, 40 ug (Total Protein)

### ELISA and Immunoblot Analysis of Guinea Pig Sera

Guinea pig serum samples were assessed for induction of specific HA antibody responses using a standard ELISA method. Briefly, Nunc Maxisorb flat-bottom 96-well plates were coated overnight with 0.1 mL/well containing 1.5 micrograms of full-length A/Wyoming/03/2003 HA protein (Protein Science Corporation). Plates were blocked with 10% nonfat dried milk in PBS for 2 h at 37°C. Serum samples were serially diluted in 1% milk solution and 100 microliter aliquots were tested for binding to antigen in triplicate. After 1 h incubation at 37°C, the plates were washed in PBS containing 0.1% Tween-20 (PBS-T) and probed with a peroxidase-conjugated goat anti-guinea pig total IgG antibody (Kirkegaard & Perry Laboratories, Inc., Gaithersburg, MD, KPL, 1:1000) for 1 h. After additional washes, bound conjugates were quantitated by the addition of tetramethylbenzidine (TMB) substrate (KPL) for 90 sec, followed by an equal volume of 0.1N sulphuric acid. Plates were read at 450 nm and mean values of triplicate wells were calculated. Plate backgrounds were determined from antigen-coated wells detected with secondary antibody only. ELISA extinction titers were calculated as the maximum serum dilutions that resulted in a signal that exceeded a value that was three times plate background (approximately 0.15 OD units). Mean values with error bars equal to one standard deviation of the triplicate were graphed as a function of time over the course of the study.

The specificity of immune responses to HA protein was assessed by Western blot analysis. Samples containing 30-50 ng of full-length recombinant A/Wyoming/03/2003 HA protein (Protein Sciences, Inc.) were electrophoresed in 4-20% Tris-Glycine gels (Invitrogen) and transferred to nitrocellulose membrane. The membrane was cut such that each replicate lane was in a single strip, blocked in a solution of 10% nonfat dried milk in PBS, and probed with sera from immunized and infected guinea pigs. After washing in PBS-T, the strips were detected with peroxidase-conjugated goat anti-Guinea pig antibody, washed again, developed with West Pico Chemiluminescent Substrate (Pierce). The blot was exposed to X-ray film and images of the strips assembled for comparison.

### Hemagglutination Inhibition Assay (HI)

A standard HI assay was performed in blinded fashion to assess Wyoming/03-specific neutralizing antibody levels [28]. Prior to assay, serum samples were treated with Receptor Destroying Enzyme (RDE, Denka Seiken CO LTD., Tokyo, Japan) overnight at 37°C followed by heat inactivation for 1 hour at 56°C. Two-fold dilutions of serum samples were mixed with A/Wyoming/03/2003 virus (at a concentration of 4 hemagglutination units per well) and incubated for 15 min at room temperature. 0.05 mL of a 0.5% suspension of chicken red blood cells was added and hemagglutination was assessed after 1 h, as described.

### Peptide Synthesis and Peptide Scanning (PepScan) Assay

**Figure 7:**
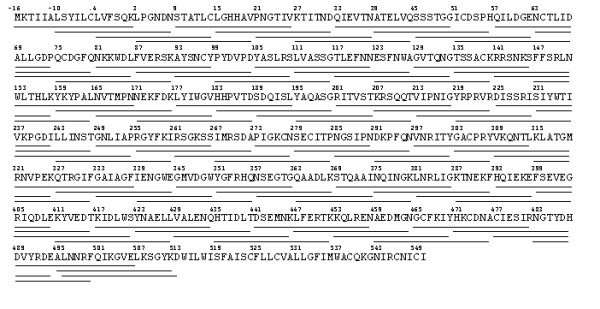
**Protein sequence of influenza A/Wyoming/03/2003 hemagglutinin glycoprotein showing location of peptides synthesized for use in PepScan analysis [GeneBank:**EU268227.1].

To map linear antibody responses, a set of overlapping peptides (Figure 7) representing amino acids -16 through 513 of the Wyoming HA glycoprotein was synthesized by Mimotopes, Inc. (Melbourne, Australia) [29]. Peptide 1 represented the amino terminus of the precursor protein, including the signal leader sequence, and was synthesized with a C-terminal linker of four residues followed by a biotin label. All other peptides were synthesized with an N-terminal linker and an N-terminal biotin. The peptides contained 18 residues and overlapped in sequence with each neighbouring peptide by 10 residues. Peptides were synthesized with a biotin conjugate to facilitate binding to streptavidin-coated microtiter plates. Figure 7 shows the overlap design of the peptides and the N-terminus number assigned to each individual peptide.

To assess immune recognition of linear epitopes, peptides were bound to plates and tested for reactivity to serum samples. Briefly, 0.1 mL of a 4 microgram/mL solution of streptavidin (Promega) was introduced into each well of Nunc Maxisorp plates and allowed to evaporate overnight at 25°C. The plates were washed ten times with PBS-T, blocked for 2 h with PBS-T and evacuated. For each peptide, 0.1 mL of a solution, adjusted to 20 microgram/mL, was placed into a well and allowed to bind overnight at 25°C, and rinsed with PBS-T. The plates were blocked overnight with 10% nonfat dried milk, at 4°C, and rinsed with PBS-T. Guinea pig serum samples were diluted in 1% milk and incubated in the wells for 2 h at 37°C. Plates were washed with PBS-T, probed with an 1 micrograms/mL solution of peroxidase-conjugated goat anti-guinea pig IgG for 1 h at 37°C, washed again, and developed with TMB solution. Bound antibody was detected in a standard plate reader using the same methods as described above for ELISA detection.

## Competing interests

The authors declare that they have no competing interests.

## Authors' contributions

RVB performed serological assays, helped prepare immunogen and data analysis, and helped write the paper; JKT performed serological assays, helped prepare immunogen, and performed data analysis; JL performed data analysis and helped design experiments; SSC performed serological assays and data analysis, provided scientific analysis, and helped write the paper; ARC helped analyze data and write the paper; PLN helped design the study, analyze data, and write the paper; GJT helped design the study, prepare recombinant protein and virus stocks, analyze data, and write the paper. All authors have read and approved the final version of this manuscript.
